# Harvard Odontological Society

**Published:** 1891-03

**Authors:** 

**Affiliations:** Harvard Odontological Society


### HARVARD ODONTOLOGICAL SOCIETY.

### DISCUSSION OF DR. STANTON'S PAPER.

(For Dr. Stanton's paper, see page 163.)

*Dr. Briggs.*—Mr. President, I recall a remark which I made at the last meeting,—viz., that the pulps which I capped were not exposed pulps; in other words, they were pulps that would have been exposed if I had removed all of the softened dentine. I dressed them with a mixture that would not harden, simply oxide of zinc mixed with an antiseptic, making as deep a layer of that as the cavity would bear, then put over that a piece of copper metal, very thin, serving, I believed, two purposes,—one, that it relieved the pulp from the pressure of the oxyphosphate, which I put on later; the other, that it had some antiseptic virtue of its own. That is the only kind of exposed pulp that I have capped. I never attempt to cap those pulps that are actually exposed, because in most cases that I have seen that "layer" gets injured, and the result is the death at some future time of the pulp, either through the formation of pulp-stones or inflammation of that organ. In some cases death may not take place until after five or ten years have passed. The trouble is then generally very serious and hard to control. In my opinion, the better way, if the tooth be fully developed at the time, is to destroy the pulp, and then make a successful filling of the root, and you will then have a very serviceable tooth. It is a very interesting point that Dr. Stanton has brought out.

*Dr. Taft.*—I should like to ask Dr. Stanton if he knows whether, in an inflammatory condition of the pulp, the cells of the odontoblastic layer take on a different appearance from what they have in a normal condition?

*Dr. Stanton.*—That is an extremely difficult question to answer. When you consider that the odontoblasts have to be magnified from eighteen hundred to three thousand diameters in order to be clearly distinguished, it is evidently very difficult to note any change of appearance. Again, it is likely that in the preparation they will be destroyed.

*Dr. Taft.*—Then, how do you know that those cells are destroyed when anything of an escharotic nature is put on? Isn't it merely theoretical knowledge?

*Dr. Stanton.*—Possibly, to a certain extent,—but inasmuch as all thoroughly exposed pulps are destroyed, it is fair to presume that this is because the odontoblasts cannot perform their work. Perhaps I do not make the point very clear, since my paper was hurriedly written, but my idea is that the irritation of deep cavities with large fillings, and the pressure of clasps, such as are used for the correction of irregularities, or as supports in bridge-work, do, in time, paralyze the odontoblastic layer and prevent a subsequent deposit of lime; and it is, furthermore, fair to presume that, as soon as the functions are interfered with so that they cannot free themselves of the lime, we get the destruction of the pulp through the formation of pulp-stones or from inflammation. We all know that when a pulp is inflamed it swells. If there is no room to allow for that swelling, the pulp and nerve become constricted at the apex and it is very easy to see what the result will be. A microscopical examination of the pulp shows that it is little more than embryonic tissue, the only regular organizations found are these odontoblasts on the surface and the circulating blood-vessels. There are no lymphatics in the pulp, you will remember,—in fact, it is the only tissue in the body that has no lymphatics; that being the case, there is no aid to the repair of injured tissue. And then, if you destroy the layer of odontoblasts, there is nothing underneath to reproduce them as there is in the case of the skin.

*Dr. Briggs.*—It goes without saying that this layer is destroyed, or at least deeply injured, when we apply escharotics to it. The treatment is certainly not conducive to the repair of injured tissue.

*Dr. Stanton.*—The fibril which enters the dentine is quite as strong as the other two which extend, the one to the deeper tissue of the pulp, the other to the neighboring odontoblasts, and in removing that softened tissue, of which Dr. Briggs speaks, you are likely to remove the layer of odontoblasts with the layer of dentine, because you cannot discern it with the naked eye; that being the case, you have destroyed the only organ of the pulp that can restore it to its healthy condition.

*Dr. Hitchcock.*—I should like to ask Dr. Briggs if an escharotic will work through and destroy the odontoblasts when used over that softened tissue?

*Dr. Briggs.*—I grant you there is a possibility of its working through provided the layer is too thin.

*Dr. Hitchcock.*—Then you would leave a fairly thick layer of that softened dentine?

*Dr. Briggs.*—I should consider all those things as treatments and should not leave them indefinitely. I should look upon them as something having the nature of a promissory note, to be endorsed in the course of a year at most, the dressing is all to be removed, and then a little more of the dentine taken away if it still is soft, the tooth dressed again in the same manner, and left for another year or two; after the second dressing, if everything seemed to be in as good condition as when I first saw the tooth, I would put in a permanent filling.

*Dr. Taft.*—The inflammatory condition being a diseased condition, the cells of the odontoblastic layer must undergo changes as in other tissues. I admit that this layer of cells is destroyed under cappings of an escharotic nature, but I also believe that it is destroyed under cappings which are not of that nature.

*Dr. Stanton.*—If a pulp has been irritated to the extent of sloughing, the result should be the same as in other tissues. If an irritation of the skin, even though it be superficial, should be sufficient to cause sloughing, the cells there are destroyed. It is an extremely fine point,—you might almost call it hypothetical, but it is not an unreasonable one.

*Dr. Taft.*—There seems to be great diversity in pulps as regards the way in which they will recover after they have been capped. A patient came to my office the other day, and referring to one of the bicuspids, which had been treated some time ago by a prominent dentist, remarked that when it was capped the pulp bled very profusely, but that pulp is alive to-day under the capping and has been perfectly comfortable ever since it was capped. I rarely attempt to cap a pulp that has been bleeding, and if I do it is after the hemorrhage has somewhat abated. Why shouldn't the odontoblastic layer in this case be destroyed almost immediately? It must have been in a high state of irritation.

*Dr. Stanton.*—Not necessarily. There are many wounds which heal without the slightest irritation and leave no scar whatever? There is a difference between a wound produced by a sharp, clean cut, and one which is the result of the diseased function of any organ. Some pulps have a greater power of recovery than others, just as there are persons whose flesh heals with wonderful rapidity and ease.

*Dr. Briggs.*—I think Dr. Smith said at the last meeting, Mr. President, that the best class of cases to treat were accidental exposures. He said there was no excuse for making them, but I believe some of the members said they did occur. Speaking of the difference in the recuperative power of pulps, some pulps do not give the cry when they are dying that others do. They seem to have the power of repressing their complaint, and a pulp may be dying slowly and surely, and it is not until it is dead and begun to slough, and we have an alveolar abscess, that signs of its death appear.

*Dr. Hitchcock.*—At the school Dr. Moffatt instructed us to perforate the pulp, letting out a drop or two of blood to relieve the inflammation, the chances being far more favorable for its recovery. The case which Dr. Taft speaks about is one of those which was benefited by the letting of blood.

*Dr. Taft.*—Yes, but you rarely have inflammation with a freshly-exposed pulp. Why should there be any need of perforating a pulp where there has been no inflammation?

*Dr. Hitchcock.*—Simply working over the tooth is likely to produce inflammation. You can realize this in operations requiring considerable excavation, where the cavity extends near the pulp, as it often causes soreness.

*Dr. Gillett.*—The case which Dr. Taft cites of a bleeding pulp being capped and, on being examined a year afterwards, found to be apparently performing its functions, recalled to my mind several cases of this kind, and I will mention one in particular, which will serve to illustrate the condition that a pulp may get into and still be apparently healthy. A lady under my care for the past four years has had for all of that time in a left lower molar a gutta-percha filling, which has been gradually wearing out. Quite lately I concluded that the filling was so much worn as to need repairing, so I re-excavated the cavity. As I removed the last of the filling a drop of pus spurted out from the bottom of the cavity; I was surprised, as I had not expected to find any trouble whatever. That filling had been in the mouth for at least five years. The pulp was evidently exposed at the time the filling was made, but the tooth has been comfortable, has given no trouble whatever during the time it has been under my care, and the pulp was apparently a healthy one. On looking into the cavity, after removing the filling, I could see that beating or throbbing that you occasionally find in a violently-inflamed pulp, and it gave every indication of being badly diseased. There was an absence of pain about it that you sometimes meet with in those cases. I mention this as an illustration of what irritation a pulp will bear and go on apparently performing its normal functions, but still becoming more and more diseased, while to any examination which can be made short of actually exposing it, it is apparently as healthy as any other pulp.

*Dr. Upham.*—Some writers have stated that the pulp is not only a formative organ, but is also destructive,—that is, the pulp, through the fibrils of its odontoblasts removes, by osmosis, lime-salts from the dentine. I should like to ask Dr. Stanton the function of the odontoblasts in this respect.

*Dr. Stanton.*—My paper is very likely to be criticised, and in all probability will be in the matter of my reference to the layer of odontoblasts as a membrane. Some writers deny that it is or can be classed as such. I have never read of the statement that Dr. Upham has just made, and therefore do not feel prepared to answer the question intelligently. I should like to ask him if he means that this is supposed to occur in a normal, healthy tooth?

*Dr. Upham.*—The statement applies to a state of general malnutrition induced by illness, disease, a low vitality. The teeth are said to be robbed of their constituent lime-salts, rendering them more liable to decay. I do not think this theory a true one, but do think, in view of the facts shown by most recent demonstrations, that the enemies immediately causing decay of the teeth are to be found in their environment.

*Dr. Stanton.*—The pulp is, to a great extent, the remains of an embryonic organ; by another stretch of the imagination you may regard the odontoblastic cells as the remnants of an embryonic cell. It is well known that in the formation of the adult tissue all over the body there is a return to the embryonic state before the permanent condition is formed; in other words, there are embryonic cells which, to a certain extent, go to the formation of tissue; but, before they reach perfection, there is a reaction, and these cells, instead of attaining completeness, take on a reformative action and proceed to form the adult tissue until the type limitation has been reached. Now, it is perfectly possible that the odontoblastic cells may be of this nature. It is perfectly well known that the osteoblasts, or bone-formers, will be converted into osteoclasts, or bone-destroyers, under certain conditions; that is, the very cells that are the depositors of bone will be converted into the cells that absorb the bone-tissue in case anything interferes with their action. In a normal condition I doubt very much if that can be so. The younger the tooth is, the larger is the pulp; the farther away you get from the pulp, the smaller is the fibril, until ultimately you have no fibrils whatever, and the pulp, instead of destroying the dentine by removing the lime-salts, becomes entirely ossified, and there is no pulp-canal or cavity.

I will speak of an experiment which I read, which consisted of feeding a female dog, during the period of gestation, on food which contained absolutely no lime-salts, no bone-forming constituents. The dog's health remained perfectly good, and, on the birth of the pups, one of them was immediately killed and the bony structure thoroughly examined to find out if any disease had been promoted through lack of nourishment. There was none discovered whatever. Other litters of pups from the same dog were examined, and though she was continually fed on food containing no lime-salts, the pups were perfectly formed and gave no evidence of a diseased condition. Finally, the female dog was killed and every part was examined; the bones were all right except those in the spinal column. There was a very decided wasting away of the vertebræ.

*Dr. Briggs.*—It is a very interesting point, that question of the rise and fall of the tooth with regard to its quality. It seems to me that the teeth do undergo changes and at some times are harder or softer than at others. How the deterioration and restoration is produced is of course what we would all like to know. This last experiment which Dr. Stanton has spoken of is interesting because I remember that in the former doctrine there was a theory—I don't know how far it has ever been carried out—that it was possible to effect painless labor by giving the mother food containing no lime-salts, whereby it was presumed that the bony system of the child would not develop until after birth, and the child, being simply a mass of flesh and cartilage, would be born with greater ease. The experiment which Dr. Stanton cites goes to prove that the child has the first claim, and the mother's bones have to give up their lime-salts to insure its development, and if there is a dearth of this material the mother suffers most largely in that direction.

H. L. Upham, D.M.D.,
*Editor Harvard Odontological Society.*

